# ChemoSensitivity Assay Guided Metronomic Chemotherapy Is Safe and Effective for Treating Advanced Pancreatic Cancer

**DOI:** 10.3390/cancers14122906

**Published:** 2022-06-13

**Authors:** William H. Isacoff, Brandon Cooper, Andrew Bartlett, Brian McCarthy, Kenneth H. Yu

**Affiliations:** 1 Department of Medicine, David Geffen School of Medicine at University of California Los Angeles, Los Angeles, CA 90095, USA; whisacoff@earthlink.net; 2 Adera Biolabs, Germantown, MD 20876, USA; brandon.cooper@aderabio.com (B.C.); andrew.bartlett@aderabio.com (A.B.); brian.mccarthy@aderabio.com (B.M.); 3 Memorial Sloan Kettering Cancer Center, Weill Cornell Medical College, New York, NY 10065, USA

**Keywords:** metronomic chemotherapy, gene expression modeling, circulating tumor and invasive cells, pancreatic cancer

## Abstract

**Simple Summary:**

Innovative chemotherapy regimens and tools to guide therapy in advanced pancreatic cancer are greatly needed. We present results of a study combining an innovative, metronomic chemotherapy strategy together with a blood-based pharmacogenomic tool to guide effective drug therapy. This study provides proof of principle that guided, metronomic chemotherapy for treatment of pancreatic cancer is a promising approach.

**Abstract:**

Cytotoxic chemotherapy remains the mainstay of treatment for advanced pancreatic adenocarcinoma (PDAC). Emerging studies support metronomic chemotherapy (MCT) as effective, challenging established paradigms of dosing and schedules. The blood-based ChemoSensitivity Assay has been shown to predict response and survival in advanced PDAC patients treated with standard chemotherapy. The current study combines these concepts for a highly personalized treatment approach. This was a retrospective analysis; a pilot (*n* = 50) and validation cohort (*n* = 45) were studied. The ChemoSensitivity Assay was performed at baseline and during therapy; results were correlated to drugs administered and patient outcomes. MCT was administered based on the assay results at the treating physician′s discretion. Patients in the pilot cohort experienced favorable survival compared with historical controls (median overall survival (mOS) 16.8 mo). Patients whose treatment closely matched the ChemoSensitivity Assay predictions experienced longer median time on lines of therapy (5.3 vs. 3.3 mo, *p* = 0.02) and showed a trend for longer mOS (20.9 vs. 12.5 mo, *p* = 0.055) compared with those not closely matched. These findings were confirmed in the validation cohort. Overall, patients treated with MCT closely matching Assay results experienced a remarkable mOS of 27.7 mo. ChemoSensitivity profiling-guided MCT is a promising approach for personalized therapy in advanced PDAC.

## 1. Introduction

Pancreatic ductal adenocarcinoma (PDAC) remains a lethal malignancy for most patients. In 2022, an estimated 62,210 new cases will be diagnosed, resulting in 49,830 deaths within the US, thus ranking it the third leading cause of cancer-related mortality [1]. Unfortunately, most patients present with advanced disease requiring systemic drug therapy. Despite the introduction of newer chemotherapeutic regimens, benefit in overall survival has been disappointing for the treatment of advanced disease, where combination chemotherapy continues to be the mainstay of therapy. Randomized phase III trials have established several first- and second-line regimens, notably FOLFIRINOX (5-fluorouracil (5-FU), leucovorin (LV), irinotecan, and oxaliplatin) [2], gemcitabine/nab-paclitaxel (G/nab-P) [3], and 5-FU/LV/nanoliposomal irinotecan (nal-IRI) [4]. Importantly, we are without tools to predict how an individual patient will respond to a given chemotherapeutic regimen, and many patients experience toxicity with only modest benefit; median response rates in the frontline setting range from 23% to 32%, median progression-free survival from 5.5 to 6.4 months, median overall survival is less than 1 year, and 2-year survival is less than 10%. Molecular profiling has been utilized to help us better understand the biology of PDAC; however, it has proven to be of little value for defining therapeutic targets [5,6,7,8], with the exception of the PARP inhibitor, olaparib, in the small subset of patients harboring germline BRCA mutations [9]. Consequently, the development of new and innovative approaches is needed. The optimal selection of effective drugs along with safe doses and schedules remains an area that warrants further investigation. The optimal selection of current FDA-approved drugs in first- and second-line regimens is still unclear. This study explores two innovative interventions to address these unmet needs.

Administration of proven cytotoxic chemotherapy drugs as part of novel combinations and using innovative schedules and dosing is an increasingly attractive approach. Combination chemotherapy is clearly more effective in this disease compared with a single-agent approach, as confirmed in the pivotal randomized phase III trials showing the superiority of both FOLFIRINOX and G/nab-P compared to single-agent gemcitabine, and 5-FU/LV/nal-IRI compared to single-agent 5-FU. More recent studies have demonstrated the promising activity of other chemotherapy combinations, such as adding cisplatin to G/nab-P [10] and even a four-drug regimen adding capecitabine to cisplatin/G/nab-P [11]. However, combination regimens, together with a longstanding approach of dosing drugs at the maximum tolerated dose (MTD), have resulted in regimens that push the limit of what our patients are able to tolerate and constrain the number of drugs that can be used together. Recent reports bring into question whether this approach is always necessary or ideal [12]. Metronomic chemotherapy (MCT) builds upon preclinical studies demonstrating effective tumor growth control from giving lower doses of cytotoxic drugs more frequently; furthermore, MCT strategies have been demonstrated to modulate the tumor microenvironment more effectively than standard MTD approaches [13]. We recently published a proof of principle study showing that an MCT approach combining 5-FU, LV, nab-P, oxaliplatin, and bevacizumab is active, leading to median overall survival exceeding that commonly observed [14]; this activity has been confirmed in a prospective phase I/II clinical trial [15].

Choosing the most effective drugs to combine using an MCT strategy to treat individual patients remains a challenge. A blood-based assay using gene expression profiling of circulating tumor and invasive cells, named the ChemoSensitivity Assay, has been developed to predict tumor response to a panel of cytotoxic chemotherapeutics. This assay has now been validated in a number of studies for predicting responses to standard combination chemotherapy regimens in advanced PDAC [16,17]. The current study was conducted to determine and validate the safety and efficacy of an MCT strategy, guided by the ChemoSensitivity Assay, for treatment of patients with advanced PDAC. The primary endpoints were survival and toxicity of patients treated following this approach compared to those seen historically with standard approaches.

## 2. Materials and Methods

### 2.1. Clinical Trial Design

This was a retrospective study; data from a total of 95 patients were analyzed. Fifty consecutive patients treated between 25 November 2014 and 29 November 2017 were included in the pilot cohort. Forty-five consecutive patients treated between 19 January 2018 and 10 December 2019 were included in the validation cohort. Data cutoff for events was 31 January 2021. The study was conducted in accordance with the Declaration of Helsinki, and the analysis was granted a waiver by the MSKCC Institutional Review Board (IRB) and its Ethics Committee.

All research was performed in accordance with institutional guidelines/regulations, and informed consent was obtained from all participants and/or their legal guardians. The primary objectives of these studies were median time on line of therapy and median OS in patients receiving MCT chemotherapy. Time on line of therapy was defined by the length of time a patient was treated with a specific drug combination. The secondary objective was to investigate the impact of drug matching between the chemotherapy drugs administered and the ChemoSensitivity Assay on both median time on line of therapy and mOS. Key eligibility criteria included: histological or cytological confirmation of PDAC, radiographic confirmation of American Joint Committee on Cancer (AJCC) stage IV disease and an Eastern Cooperative Oncology Group (ECOG) performance status of 0, 1, or 2. Following written informed consent and prior to initiation of chemotherapy treatment, a 6 mL blood sample was obtained in a sodium-heparinized Vacutainer tube (Becton Dickinson, Franklin Lakes, NJ, USA) from each study participant using standard clinical procedures. Additionally, blood samples were collected and processed at the time of treatment change. Results of the ChemoSensitivity Assay were disclosed to the treating physician; however, drug selection was at the physician′s discretion.

### 2.2. Metronomic Chemotherapy (MCT)

The seven chemotherapeutic agents were typically flat dosed as follows: gemcitabine 800–1000 mg IV/30 min, nab-paclitaxel 70–100 mg IV/30 min, 5-FU 200–300 mg/day for 14 days, oxaliplatin 85 mg IV/120 min, irinotecan 80–120 mg IV/90 min, mitomycin-C 10–14 mg IV bolus, and cisplatin 25–50 mg/60 min. Of these, two to four drugs were combined and administered weekly. Typically, these combinations contained 5-FU, gemcitabine, or both, with the remaining drugs added at the physician′s discretion based on clinical judgment and the results of the ChemoSensitivity Assay. The data for safety and tolerability were extracted from the medical records for patients enrolled in the pilot study and graded using the Common Terminology Criteria for Adverse Events (CTCAE) v4.0. Treatment regimens were changed primarily based on rising levels of CA 19–9, and less frequently based on disease progression seen on cross-sectional imaging. The ChemoSensitivity Assay was often performed at the time of treatment regimen change. Safety and tolerability were extracted from patient medical records for patients enrolled in the pilot study and graded using the CTCAE v4.0.

### 2.3. ChemoSensitivity Assay

Coded and deidentified samples were shipped at 4 °C overnight to Adera Biolabs (Germantown, MD, USA) for circulating tumor and invasive cell (CTIC) isolation and enriched as previously described; development of the ChemoSensitivity Assay has been described and validated in clinical trials, as previously published [16,17]. Briefly, a collagen adhesion matrix in a modified cell invasion assay was used to capture epithelial cell adhesion molecules (EPCAMs) plus invasive cells, a well-characterized approach for capturing circulating tumor cells (CTCs) [18,19,20,21]. The ChemoSensitivity Assay accurately measures the gene expression of 95 genes by quantitating mRNA levels by qPCR for each gene in circulating-tumor and invasive cells isolated from whole blood (6 mL). The seven chemotherapeutic agents modeled by the assay are gemcitabine, nab-paclitaxel, 5-FU, oxaliplatin, irinotecan, mitomycin C, and cisplatin.

### 2.4. Nearest Template Prediction

To match patient blood samples to cell line-derived drug sensitivity templates, nearest template prediction analysis was performed (previously described in [22,23,24]), with its underlying premise being that if two samples share similar expression profiles of key, relevant genes, they will have similar drug responses. We have previously published a detailed description and validation in clinical studies [16,17]. The nearest template prediction methodology was used to match chemotherapeutic templates to the profiles derived from individual patient blood samples and to sort the templates in ranked-order to obtain sensitivity values for each of the seven chemotherapeutic agents (Adera Biolabs, Germantown, MD, USA).

Individual drugs were considered a match if the nearest template prediction yielded a positive score and not a match in cases of a negative score. Drugs administered to the study participant considered a match by these criteria were tabulated (range 0–7). Drugs administered within 28 days of performing the assay were included.

### 2.5. Statistical Analysis

Survival was estimated using Kaplan–Meier analysis. Differences in survival were estimated using the logrank test and the logrank test for trend (Prism version 9.1.0, GraphPad Software, LLC., San Diego, CA, USA).

## 3. Results

### 3.1. Safety and Efficacy of Metronomic Chemotherapy Approach

In the pilot cohort, 50 patients with AJCC stage IV PDAC eligible for combination chemotherapy were included for analysis. Key patient characteristics are shown in Table 1. Patients were treated using an MCT approach, as described in the Materials and Methods section. Patient outcomes, including time on line of therapy across multiple sequential lines of therapy, safety, and survival, were recorded. The most common toxicities experienced by patients enrolled in the pilot study are shown (see Table 2). The most common grade III/IV toxicities include cytopenias, anorexia, weight loss, and stomatitis.

### 3.2. ChemoSensitivity Assay

CTICs were isolated, greater than 1.0 ng/μL of RNA was extracted, and gene expression profiling was successfully performed from a single 6 mL heparinized whole-blood sample in >99% of samples received. The rare failures can be attributed to problems in sample shipping and handling, insufficient RNA quantities, cDNA yield, or PCR well errors typically isolated to failure of a single gene to amplify or exponential algorithm failure. Assay reliability was regulated and assessed in accordance with CLIA-certified mandates for molecular analysis controls and sample consistency.

For each sample analyzed, the ChemoSensitivity Assay determines relative sensitivity to a panel of seven commonly used cytotoxic chemotherapeutics: cisplatin, 5-FU, gemcitabine, irinotecan, mitomycin-C, nab-P, and oxaliplatin. Drugs scoring ≥0 were considered relatively effective, while those scoring <0 were considered relatively ineffective. Drugs administered during a window of ±28 days from the date the assay was performed were included in the analysis. The number of drugs administered with a score ≥0 was tabulated for each patient both at baseline and at times of disease progression.

### 3.3. ChemoSensitivity Assay-Guided MCT, Pilot Cohort

**Figure 1:**
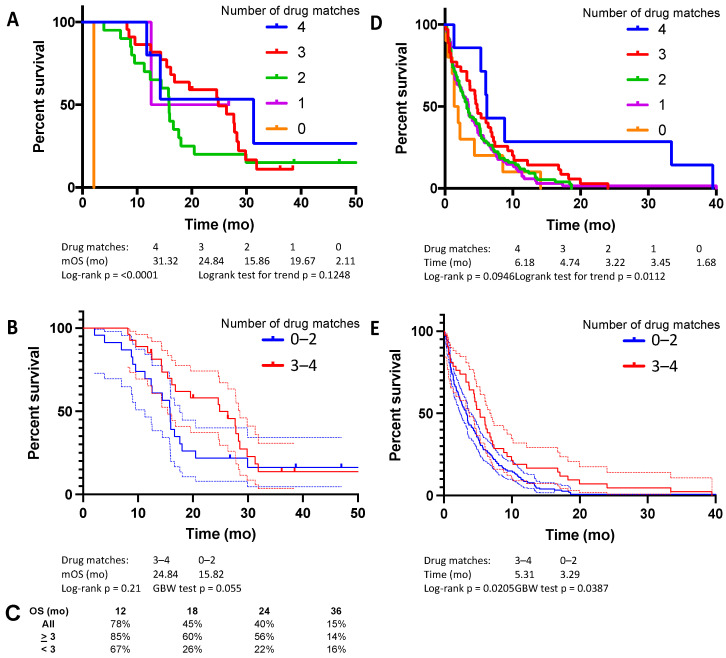
Pilot study, median OS, based on (**A**) number of drugs in frontline regimen matched to ChemoSensitivity Assay results, and (**B**) threshold, either three or more matches or two or fewer. (**C**) Percent OS at 12, 18, 24, and 36 months. Median time on line of therapy, based on (**D**) number of drugs in frontline regimen matched to the ChemoSensitivity Assay results, and (**E**) threshold, either three or more matches or two or fewer.

The median time of follow-up and overall survival for the pilot cohort were 16.7 and 16.8 months, respectively. Blood samples were collected at baseline and during treatment. Frontline treatment regimens were scored by the ChemoSensitivity Assay as follows: 1 (2%) with zero drug matches, 2 (4%) with one drug match, 20 (40%) with two drug matches, 22 (44%) with three drug matches, and 5 (10%) with four drug matches. The frequency with which these drugs were included in the frontline regimen is summarized in Table 1. Median overall survival (OS) was 31.3 mo with four matched drugs, 24.8 mo with three matched drugs, 15.9 mo with two matched drugs, 19.7 mo with one matched drug, and 2.1 mo with zero matched drugs (Figure 1A, logrank, *p* < 0.0001). Patients were divided into two groups for further analysis: those treated with a regimen that closely matched the ChemoSensitivity Assay prediction (≥3 drug matches) and those that did not (≤2 drug matches). Patients whose regimens more closely matched assay results had numerically superior median OS compared to those not closely matched, though this did not reach statistical significance (Figure 1B). Median OS was 24.8 mo (95% confidence interval (CI) 15.3 to 28.6 mo) with ≥3 drug matches and 15.8 mo (95% CI 11.2 to 17.7 mo) with ≤2 drug matches (*p* = 0.21, hazard ratio (HR) 0.66, 95% CI 0.35 to 1.26). Time on line of therapy was also measured across multiple lines of therapy and analyzed based on the ChemoSensitivity Assay results (Figure 1). Median time on line of therapy was 6.18 mo with four matched drugs, 4.7 mo with three matched drugs, 3.2 mo with two matched drugs, 3.45 mo with one matched drug, and 1.7 mo with zero matched drugs (Figure 1D, logrank test for trend, *p* = 0.0112). Separating the patients into two cohorts, median time on line of therapy was 5.3 mo (95% CI 3.8 to 6.8 mo) with ≥3 drug matches and 3.3 mo (95% CI 2.4 to 4.0 mo) with ≤2 drug matches (Figure 1E, *p* = 0.02, HR 0.69, 95% CI 0.50 to 0.94 mo). Significant differences in median OS and time on line of therapy were seen based on the number of drug matches, with numerically longer survival and time on line of therapy in patients treated with regimens more closely matched to the assay.

### 3.4. ChemoSensitivity Assay-Guided MCT, Validation Cohort

Based on the encouraging results of the pilot cohort, a validation cohort was studied. AJCC stage IV PDAC patients (*n* = 45) were analyzed (Table 1). Overall, patients in the validation cohort were treated with frontline regimens containing a higher number of matched drugs than the pilot cohort. Patient breakdown based on the ChemoSensitivity Assay results is as follows: 3 (7%) with one drug match, 7 (16%) with two drug matches, 19 (42%) with three drug matches, 10 (22%) with four drug matches, 3 (7%) with five drug matches and 1 (2%) with six drug matches. Median time of follow-up and OS for the validation cohort were 15.5 and 25.9 months, respectively.

**Figure 2:**
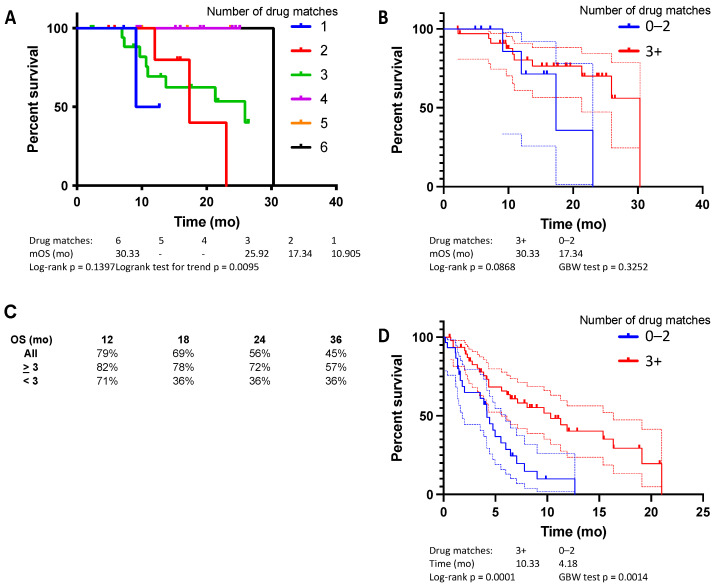
Validation study, median OS, based on (**A**) number of drugs in frontline regimen matched to the ChemoSensitivity Assay results, and (**B**) threshold, either three or more matches or two or fewer. (**C**) Percent OS at 12, 18, 24, and 36 months. (**D**) Median time on line of therapy based on number of drugs in frontline regimen matched to the ChemoSensitivity Assay results, either three or more matches or two or fewer.

Median OS was 30.3 mo with six matched drugs, not reached with five or four matched drugs, 25.9 mo with three matched drugs, 17.3 mo with two matched drugs and 10.9 mo with one matched drug (Figure 2A, logrank test for trend, *p* = 0.0095). Comparing those treated with a regimen that closely matched the ChemoSensitivity Assay prediction (≥3 drug matches) to those who were not (≤2 drug matches), we observed a benefit for patients whose regimens more closely matched assay results (Figure 2B). Median OS was 30.3 mo (95% CI 21.2 to 30.3 mo) with ≥3 drug matches and 17.3 mo (95% CI NA to 23.1 mo) with ≤2 drug matches (*p* = 0.087, HR 0.57). Time on line of therapy was also measured across multiple lines of therapy and analyzed based on the ChemoSensitivity Assay results (Figure 2D). Median time on line of therapy was 10.3 mo (95% CI 6.1 to 17.8 mo) with ≥3 drug matches and 4.2 mo (95% CI 3.7 to 7.0 mo) with ≤2 drug matches (*p* = 0.0001, HR 0.40).

**Figure 3:**
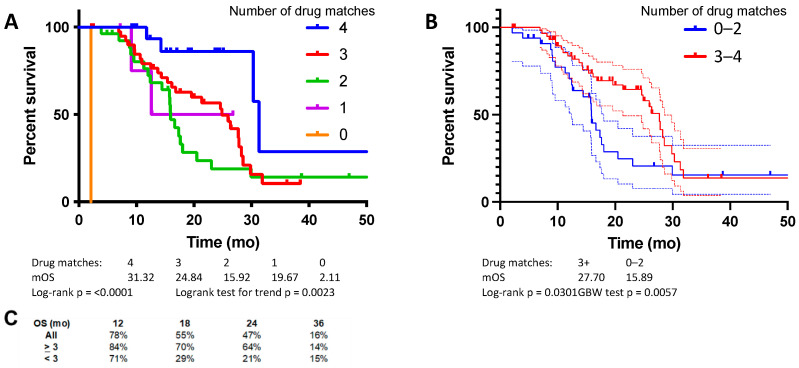
Combined analysis of pilot and validation cohorts, median OS, based on (**A**) number of drugs in frontline regimen matched to the ChemoSensitivity Assay results, and (**B**) threshold, either three or more matches or two or fewer. (**C**) Percent OS at 12, 18, 24, and 36 months.

A combined analysis of the pilot and validation cohorts demonstrated an overall median OS of 21.4 mo. Median OS was 31.3 mo with four or more matched drugs, 24.8 mo with three matched drugs, 15.9 mo with two matched drugs, 19.7 mo with one matched drug, and 2.1 mo with no matched drugs (Figure 3A, logrank test for trend, *p* = 0.002). Comparing those treated with a regimen that closely matched the ChemoSensitivity Assay prediction (≥3 drug matches) to those who were not (≤2 drug matches), there was a significant improvement in mOS for patients whose regimens more closely matched the assay results (Figure 3B). Median OS was 27.7 mo with ≥3 drug matches and 15.9 mo with ≤2 drug matches (*p* = 0.03, HR 0.57).

### 3.5. Individual Examples of ChemoSensitivity Assay-Guided MCT

Patient 18-003 presented at age 71 with PDAC metastatic to the liver. The baseline assay predicted sensitivity to gemcitabine, nab-paclitaxel, irinotecan, and mitomycin-C. Initial treatment included three of these drugs. The patient received gemcitabine, nab-paclitaxel, irinotecan, and 5-FU. Irinotecan, nab-paclitaxel, and 5-FU were individually held in certain cycles for toxicity management. Baseline CA 19-9 was minimally elevated at 46 U/mL and responded by dropping to as low as 11 U/mL after 4 months of therapy. The assay was performed again after 6 months of therapy. The assay no longer predicted sensitivity to nab-paclitaxel, while sensitivity remained for gemcitabine, irinotecan, and mitomycin-C. Treatment largely remained unchanged, although mitomycin-C was administered periodically. The most common combination used was gemcitabine, irinotecan, and nab-paclitaxel. The assay was performed again after 12 months of therapy. At this time CA 19-9 measured 13 U/mL. The assay continued to predict sensitivity for gemcitabine, irinotecan, and mitomycin-C. Treatment remained largely unchanged; however, oxaliplatin was substituted for nab-paclitaxel in alternating fashion. The assay was performed again after 15 months of therapy, with CA 19-9 rising minimally to 20 U/mL. The assay then predicted sensitivity to gemcitabine, nab-paclitaxel, and oxaliplatin. Treatment consisted of gemcitabine and nab-paclitaxel, with irinotecan alternating with oxaliplatin. CA 19-9 remained low and was measured at 12 U/mL 9 months later. At the data cut-off point, the patient remained alive and on treatment 26 months from presentation.

Patient 18-050 was a 38-year-old woman who presented with metastatic PDAC. Baseline CA 19-9 was elevated at 1331 U/mL. The baseline assay predicted sensitivity to gemcitabine, irinotecan, and mitomycin-C. She began MCT treatment with weekly gemcitabine, nab-paclitaxel, 5-FU, and oxaliplatin. The assay predicted sensitivity to only one of these drugs, gemcitabine. The patient experienced 1.8 months on this line of therapy. Treatment was then changed to gemcitabine, nab-paclitaxel, 5-FU, and irinotecan. Over the coming months, nab-paclitaxel and 5-FU were discontinued and mitomycin and oxaliplatin were added. CA 19-9 responded, dropping to 27 U/mL. After seven months, CA 19-9 rose to 14,614 U/mL. The assay was repeated, now showing sensitivity to gemcitabine, irinotecan, nab-paclitaxel, and oxaliplatin. Treatment was changed to gemcitabine, nab-paclitaxel, and irinotecan; however, the patient declined clinically and passed 9.1 months after starting treatment.

## 4. Discussion

Chemotherapy for many patients with advanced PDAC is ineffective and associated with significant toxicity. Over the last decade, a number of cytotoxic chemotherapeutic agents for treating PDAC have been developed; however, the optimal approach for dosing, scheduling, and choosing drugs for patients remains an open area of investigation. The current study combines two promising and innovative approaches to address these issues: metronomic dosing and scheduling of active agents guided by the innovative blood-based ChemoSensitivity Assay.

Administration of cytotoxic chemotherapy at the MTD can result in significant toxicity for patients. The higher drug doses used in MTD approaches result in long time intervals between treatments, which have been hypothesized to allow for outgrowth of chemotherapy-resistant clones, leading to treatment failure [25]. By using significantly lower doses, MCT not only allows for more frequent drug administration and overall improved tolerability, but there may be biological advantages as well (reviewed in [26]), including antiangiogenic effects [27,28] and targeting of PDAC stem cells [29]. In a recent retrospective study of advanced PDAC, MCT combining low-dose continuous 5-FU, LV, nab-P, oxaliplatin, and bevacizumab treatment was well-tolerated and median OS was 19 months [14].

The current study builds upon our prior work, integrating an increasing number of active agents into MCT regimens. Taken together, patients treated in the pilot and validation studies experienced favorable median OS of 21.4 mo, despite all patients presenting with metastatic disease. A key factor was integration of the ChemoSensitivity Assay to guide drug choice. The current study demonstrates a clear trend toward benefit, with regard to both time on line of therapy and median OS, based on the number of drugs administered that were predicted by the assay to be effective. Patients in the most favorable group, who received three or more drugs predicted by the assay to be effective in their frontline regimen, experienced a median OS of 27.7 mo. Furthermore, 47% and 16% of all patients treated using the MCT approach were alive at 24 and 36 months, respectively. These results compare favorably to recent historical controls. Similar patients treated with FOLFIRINOX in the randomized phase III PRODIGE 4/ACCORD 11 study experienced a median OS of 11.1 mo [2]; those treated with gemcitabine and nab-paclitaxel in the randomized phase III MPACT trial experienced a median OS of 8.5 mo. The 24-month survival for FOLFIRINOX and gemcitabine/nab-paclitaxel was 5% and 9%, respectively, with 36-month survival of 1% and 0%, respectively.

The ChemoSensitivity Assay has previously been shown in numerous studies to be a convenient and effective blood-based assay for predicting multiple lines of effective standard therapies for the treatment of advanced PDAC [16,17]. In the most recent of these studies, the optimal regimen, either FOLFIRINOX or gemcitabine/nab-paclitaxel, was predicted by the assay based on the single highest scoring drug. The current set of pilot and validation MCT cohorts is the first to fully leverage the individual drug predictions of the assay for studying the effectiveness of personalized treatment regimens. The assay was effective for predicting drug therapies not only in the frontline setting but also in subsequent lines. The MCT approach was also well-tolerated, with adverse event profiles that compared favorably to approved combination regimens.

The ChemoSensitivity Assay has several important advantages, including the utilization of a small volume of peripheral blood and providing a clinically useful tool that can be used to determine optimal chemotherapeutic drugs within a clinically meaningful timeframe and longitudinally with sequential profiling. As previously described, the assay is based upon an innovative gene expression algorithm derived from a diverse set of cancer cell lines [16,30]. Cells profiled in the assay are termed circulating tumor and invasive cells (CTICs) due to the heterogeneous population of cells captured. Prior work has shown that only between 0.03 to 0.07% of the cells isolated represent classic EPCAM(+) CTCs [31]. Importantly, we have now conducted multiple studies showing that profiling this mixed CTIC cell population is a powerful predictor of tumor drug sensitivity, translating to improved drug responses and survival.

The assay is based on profiling of 95 genes, as previously described [16,17]. These genes play important roles in the transport of drugs and other molecules. These include, but are not limited to, *ABC* superfamily gene members and solute carrier (*SLC*) genes. Many of these genes have established roles as active drug transporters and have been shown to reduce accumulation of chemotherapy drugs within resistant cancer cells [32,33,34,35,36]. The expression of some genes in PDAC tumor tissue, including *SLC28A1* and *SLC29A1*, has been associated with gemcitabine response [37]. Limitations of the current study include its retrospective nature and relatively small size. Furthermore, all patients were treated at a single center, introducing the possibility of selection bias. The exact drug combinations administered varied greatly from patient to patient and evolved over time as treating physicians became more familiar with the ChemoSensitivity Assay. Formalizing drug combinations and optimizing drug selection and schedules responsive to the ChemoSensitivity Assay results are needed such that this approach can be widely generalized. A larger, prospective, guided study is warranted and currently in development to fully validate the ChemoSensitivity Assay-guided MCT approach described here. Validation of the assay signature against existing gene-expression databases could be performed, and an ongoing trial is addressing this cross-platform comparison prospectively (*ClinicalTrials.gov* identifier: NCT04469556). Further preclinical studies to optimize and understand the biological mechanisms of this approach are also warranted.

## 5. Conclusions

We demonstrate that metronomic dosing and schedules combining active agents at low doses are both safe and effective. Furthermore, we show that using the innovative blood-based ChemoSensitivity Assay to guide drug selection significantly improves overall survival and compares favorably to historical standards.

## Figures and Tables

**Table 1:** Patient characteristics.

Characteristics	Pilot Cohort	Validation Cohort
Total, N	50	45
Age, Y, median	62	74
Gender, N (%)		
Male	26 (52)	28 (62)
Female	24 (48)	17 (38)
Stage		
IV	50 (100)	45 (100)
Disease location (%)		
Locally recurrent	15 (30)	10 (22)
Liver	33 (66)	24 (53)
Lung	7 (14)	3 (7)
Peritoneum	2 (4)	11 (24)
Frontline regimen contains		
5-FU	43 (86)	25 (56)
Nab-paclitaxel	31 (62)	34 (76)
Irinotecan	23 (46)	15 (33)
Oxaliplatin	21 (42)	34 (76)
Gemcitabine	20 (40)	33 (73)
Mitomycin-C	16 (32)	8 (18)
Cisplatin	11 (22)	3 (7)

**Table 2:** Adverse events, pilot cohort.

Toxicity	Grade (%)			
	I	II	III	IV
Fatigue	18 (38)	26 (55)	3 (6)	0 (0)
Anorexia	24 (51)	12 (26)	7 (15)	0 (0)
Drug allergy	0 (0)	5 (11)	3 (6)	0 (0)
Nausea	21 (45)	20 (43)	2 (4)	0 (0)
Vomiting	8 (17)	13 (28)	2 (4)	0 (0)
Stomatitis	13 (28)	7 (15)	6 (13)	0 (0)
Diarrhea	9 (19)	19 (40)	4 (9)	0 (0)
Weight loss	23 (49)	2 (4)	6 (13)	0 (0)
Hypertension	0 (0)	10 (21)	6 (13)	0 (0)
Renal insufficiency	0 (0)	4 (9)	3 (6)	4 (9) *
Neuropathy	12 (26)	11 (23)	2 (4)	1 (2)
Hearing loss	2 (4)	0 (0)	1 (2)	0 (0)
Anemia	7 (15)	20 (43)	16 (34)	2 (4)
Neutropenia	9 (19)	10 (21)	7 (15)	11 (23)
Thrombocytopenia	9 (19)	12 (26)	9 (19)	8 (17)

* Dialysis.

## Data Availability

The data that support the findings of this study are available from Adera Biolabs, Germantown, MD, but restrictions may apply to the availability of these data, which were used under license for the current study and so are not publicly available. Data are, however, available from the authors upon reasonable request and with permission from Adera Biolabs, Germantown, MD.
